# Low and seasonal malaria transmission in the middle Senegal River basin: identification and characteristics of *Anopheles *vectors

**DOI:** 10.1186/1756-3305-5-21

**Published:** 2012-01-23

**Authors:** Mamadou O Ndiath, Jean-Biram Sarr, Lobna Gaayeb, Catherine Mazenot, Seynabou Sougoufara, Lassana Konate, Franck Remoue, Emmanuel Hermann, Jean-francois Trape, Gilles Riveau, Cheikh Sokhna

**Affiliations:** 1 Institut de Recherche pour le Développement, UMR 198 URMITE Campus international de Hann, IRD BP 1386 CP 18524 Dakar, Sénégal; 2 Institut de Recherche pour le Développement, UMR 224 MIVEGEC, 911 avenue Agropolis- BP 64501, F-34394 Montpellier, France; 3 CIIL, Inserm U1019, CNRS UMR 8204, Université Lille Nord de France, Institut Pasteur de Lille, 1 rue du Pr. Calmette 59019 Lille cedex; 4 Université Cheikh Anta Diop de Dakar, Département de Biologie Animale, BP 5005 Dakar, Sénégal; 5 Institut de Recherche pour le Développement, UMR 224 MIVEGEC, Maladies infectieuses et vecteurs (UM1-UM2-CNRS 5290-IRD224), CREC 01BP 4414, Cotonou, Bénin; 6 Centre Espoir pour la Santé, Laboratoire de Recherches Médicales BP: 226, Saint Louis Sénégal

**Keywords:** Malaria transmission, *Anopheles arabiensis*, *Plasmodium *infection, KDR mutation, Senegal River basin.

## Abstract

**Background:**

During the last decades two dams were constructed along the Senegal River. These intensified the practice of agriculture along the river valley basin. We conducted a study to assess malaria vector diversity, dynamics and malaria transmission in the area.

**Methods:**

A cross-sectional entomological study was performed in September 2008 in 20 villages of the middle Senegal River valley to evaluate the variations of *Anopheles *density according to local environment. A longitudinal study was performed, from October 2008 to January 2010, in 5 selected villages, to study seasonal variations of malaria transmission.

**Results:**

Among malaria vectors, 72.34% of specimens collected were *An. arabiensis*, 5.28% *An. gambiae *of the S molecular form, 3.26% M form, 12.90% *An. pharoensis*, 4.70% *An. ziemanni*, 1.48% *An. funestus *and 0.04% *An. wellcomei*. *Anopheles *density varied according to village location. It ranged from 0 to 21.4 *Anopheles*/room/day and was significantly correlated with the distance to the nearest ditch water but not to the river.

Seasonal variations of *Anopheles *density and variety were observed with higher human biting rates during the rainy season (8.28 and 7.55 *Anopheles *bite/man/night in October 2008 and 2009 respectively). Transmission was low and limited to the rainy season (0.05 and 0.06 infected bite/man/night in October 2008 and 2009 respectively). During the rainy season, the endophagous rate was lower, the anthropophagic rate higher and L1014F kdr frequency higher.

**Conclusions:**

Malaria vectors are present at low-moderate density in the middle Senegal River basin with *An. arabiensis *as the predominant species. Other potential vectors are *An. gambiae *M and S form and *An. funestus*. Nonetheless, malaria transmission was extremely low and seasonal.

## Background

In many African countries, food self-sufficiency is a goal that favors the development of irrigated areas. This strategy requires the management of water resources and the implementation of new hydro-agricultural arrangements. Northern Senegal (Senegal River basin) is located in a Sahelian area with low rainfall concentrated during the short rainy season between July and October (around 200 mm/year in 2008). During the 1980s', important development programs have been implemented including the construction of two dams on the Senegal River [1]. Following these developments, irrigated areas have been enlarged and rice culture expanded.

These ecological changes have largely promoted an increase in water-related diseases such as malaria, Rift Valley fever and schistosomiasis, [2,3]. In particular, changes in malaria vector densities were reported [4-6]. *An. gambiae s.l*. and *An. funestus *used to be the two main malaria vectors in this region; however, the latter disappeared after the 1970s' droughts [7]. In a study conducted in 1999, the first after dam construction, *An. funestus *was again reported as the dominant species in the area [8]. The environmental changes associated with water development projects were suspected to be responsible for having created favourable conditions for the reestablishment of *An. funestus*.

After the implementation of the dams, water for irrigation was available; food crop was promoted in this area. Agricultural techniques changed and the use of insecticide increased. As in other African countries, this has contributed to the selection of resistant mosquito strains [9]. The presence of kdr mutation genotype, which has been recognized to be related to DDT and pyrethroid resistance [10] has been detected in various areas in Senegal [11].

This study was undertaken as part of the larger "*AnoPal-AnoVac" *study that aimed to determine whether exposure to *Anopheles *mosquitoes could alter humoral and cellular immune responses in children living in an area where malaria is present. In order to select study participants, an evaluation of exposure to *Anopheles *bites was undertaken in 20 villages. An entomological study was performed to provide up to date entomological information, describe the anophelian populations, their potential role as a vector of malaria and identify environmental factors affecting mosquito density.

## Methods

### Study area

**Figure 1:**
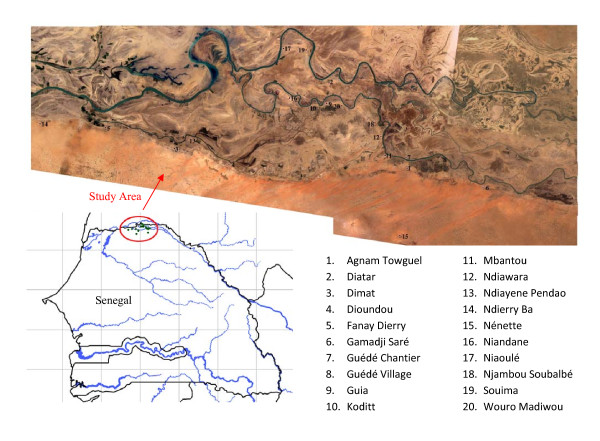
**Map of the studied area in the middle Senegal River basin**.

The study was carried out in Northern Senegal, in 20 villages of the Senegal River basin (Agnam Towguel, Diatar, Dimat, Dioundou, Fanay Dierry, Gamadji Saré, Guédé Chantier, Guédé Village, Guia, Koditt, Mbantou, Ndiawara, Ndiayene Pendao, Ndierry Ba, Nénette, Niandane, Niaoulé, Njambou Soubalbé, Souima, Wouro Madiwou, Figure 1) representing an area of 70 km east-west and 30 km north-south.

In this region, the climate is sahelian, with annual rainfall between July and October (340 mm in 2009). The mean temperature range between 20°C and 30°C during the cool season (November to February) and 25°C to 38°C during the warm season (March to October). Vegetation is sparse, with few trees in and around villages. Domestic animals including cows, horses, donkeys, sheep and goats are usually bred in open enclosures near houses. The houses have mud walls with grass thatch or corrugated iron roofs.

In all villages, GPS coordinates of mosquitoes collection sites were recorded as well as coordinates of the nearest running water (Senegal River or large irrigation canal), ditch water (rice culture, brick production or cattle watering place) and temporary pools in flooded areas. The mean distance between sampling sites and water source were calculated. Villages were classified according to their geographical situation in Walo (flood areas: Agnam Towguel, Diatar, Dimat, Dioundou, Guédé Chantier, Guédé Village, Guia, Koditt, Mbantou, Niandane, Niaoulé, Wouro Madiwou) or Dierry (dry areas: Fanay Dierry, Guamadji Sarré, Ndiawara, Ndiayene Pendao, Ndierry Ba, Nénette, Njambou Soubalbé, Souima).

Prior to the study, permission was sought from the village elders; village meetings were conducted to explain the purpose of the study and participation requested. Verbal consent was obtained to collect mosquitoes from houses. Ethical approval was given by the Senegalese National Ethics Committee.

### Mosquito sampling

Night landing catches (NLC) were performed from 19:00 to 07:00 hours. Four adult volunteer collectors were positioned at two different sites in each village. Two collected mosquitoes indoors and two outdoors. Pyrethrum spray catches (PSC) were conducted in five randomly selected rooms among those not having used any form of insecticide or repellent during the previous week and being different from those used for NLC. Deltamethrin (Yotox^®^) was sprayed inside the closed rooms for 30-45 seconds. After 10 minutes, dead or immobilized mosquitoes were collected. *Anopheles *species were identified using morphological characteristics according to identification rules [12]. Human Biting Rate (HBR) was estimated by the number of bites per person per night sampled by NLC.

### Study design

The cross-sectional study was performed in September 2008 (in the middle of rainy season). PSC mosquitoes sampling was conducted for one day in 20/20 villages and HLC for one night in 5/20 villages.

For the longitudinal study, HLC were performed in five selected villages (Agnam Towguel, Fanay Dierry, Guédé Village, Ndiayene Pendao, Niandane) for two non consecutive nights and PSC for one day, both in October 2008, January 2009, May 2009, October 2009 and January 2010.

Endophagous rates were calculated as the proportion of the number of mosquitoes captured indoor to the total number of mosquitoes captured by HLC.

### Laboratory analyses

Blood fed females captured by PSC had their blood meal squashed on Whatman No. 1 filter papers and tested by enzyme-linked immunosorbent assay (ELISA) to identify whether blood was of bovine, ovine, caprin (sheep and goat), equine (horse and donkey), or chicken origin [13]. Anthropophilic rate was calculated as proportion of blood-fed mosquitoes that had exclusively fed on human blood. All mosquitoes belonging to the *An. gambiae *complex were identified using the RFLP-PCR method using *HhaI *restriction enzyme [14]. The expression of Circumsporozoite Protein (CSP) was determined by performing ELISA with monoclonal antibodies against *Plasmodium falciparum *on the crushed head and thorax [15]. Infection rate was calculated as the proportion of positive mosquitoes to the total number of malaria vectors. Entomological inoculation rate (EIR) was calculated as the infection rate multiplied by HBR calculated for vectors species (*An. gambiae *s.l. and *An. funestus*). Detection of L1014F kdr mutation in *An. gambiae *s.l. was performed by PCR [16]. Both genotype and allelic frequencies were calculated.

### Statistical analyses of data

Qualitative data were compared using Pearson Chi^2 ^or Fisher exact test and quantitative data by non parametric tests (Spearman correlation, Kruskal-Wallis and Mann Whitney test) Statistical analyses were performed with Stata ^® ^10.1. A p value of 0.05 or less was considered as significant.

## Results

From September 2008 to January 2010, 2426 *Anopheles *specimens were collected. Among these, 446 (18.38%) were collected feeding on humans outdoors, 413 (17.02%) were collected feeding on humans indoors and 1567 (64.59%) were collected resting indoors. 1755 (72.34%) were identified as *An. arabiensis*, 128 (5.28%) *An. gambiae *molecular form S, 79 (3.26%) molecular form M, 313 (12.90%) *An. pharoensis*, 114 (4.70%) *An. ziemanni*, 36 (1.48%) *An. funestus *and 1 (0.04%) *An. wellcomei*. All species were captured by HLC and all but *An. wellcomei *and *An. ziemanni *by PSC. *An. arabiensis *were present in all but one village (19), *An. pharoensis *in only 9, *An. ziemanni *and *An. funestus *in 6, *An. gambiae *molecular form M in 5, *An. gambiae *form S and *An. wellcomei *in 1.

### Cross-sectional study

**Figure 2:**
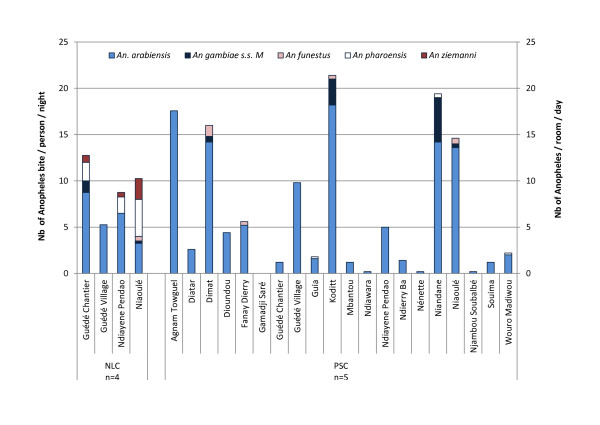
***Anopheles *density in the 20 villages of the study area during the cross-sectional study**. *Anopheles *density measured in September 2008 by Night Landing Catches NLC (number of *Anopheles*/person/night, n = 5 person/night) and by Pyrethrum Spray Catches PSC (number of *Anopheles */room/day, n = 4 room/day) in all villages according to the different species studied *Anopheles funestus, Anopheles gambiae s.s*. molecular form M, *An. arabiensis, Anopheles pharoensis and Anopheles ziemanni)*.

The presence and relative density of each species in the 20 villages is presented on Figure 2.

Since the density was highly variable between the villages, ranging from 0 in Gamadji Saré to 21.4 in Koditt, its environmental determinants were studied. The distance between sampling sites in the village and river ranged from 0.11 to 11.08 km (mean 0.62 ± 1.6) and was not correlated with Human Biting Rate (HBR) (Spearman rho -0.05, p = 0.83). The distance to ditch water ranged from 0.14 to 3.9 km and was significantly correlated with HBR (Spearman rho -0.45, p < 0.05). In the villages classified as Walo (flooded areas), HBR was significantly higher than in villages classified as Dierry (dry area) (9.3 ± 2.3 vs. 1.7 ± 0.8, Mann-Whitney test p = 0.01).

### Longitudinal study

During the 16 month study period, the density, nature and relative proportion of *Anopheles *species collected varied according to the season (Figure 2). *An. pharoensis *and *An. ziemanni *were collected in all seasons, *An. arabiensis *was collected all year round except in June 2009. *An. gambiae *M and S forms were found only during the rainy season in October 2008 and October 2009 and *An. funestus *was only collected during the rainy season in October 2009. HBR varied considerably according to the season (Figure 2); higher values were observed during the rainy season (8.28 *Anopheles *bite/man/night in October 2008) and (7.55 *Anopheles *bite/man/night in October 2009) and lower values during the dry season (0.30 in January 2009 and 0.65 in January 2010).

**Figure 3:**
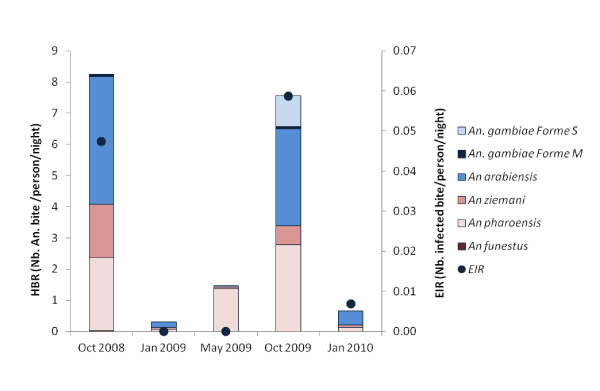
***Anopheles *density and malaria transmission from October 2008 to January 2010**. Human biting rate (HBR, number of *Anopheles *bite/person/night) according to the different *Anopheles *species and entomologic inoculation rate (EIR, number of infected bite/person/night) during the longitudinal study, n = 40 person/night each month.

Specimens infected by *Plasmodium *were detected only among mosquitoes belonging to *An. gambiae s.l*.: 10/1055 *An. arabiensis*, 1/30 *An. gambiae *molecular form M and 3/128 for the S form (Table 1). Infection rates did not vary significantly among the different species (Fisher exact p = 0.09). Among malaria vector species, global infection rate was 1.13%. Circumsporozoite Protein (CSP) positive mosquitoes were mainly sampled during the rainy season but no significant variation in infection rate was observed (Fisher exact p = 0.7, Table 1). Transmission was seasonal with 0.047 and 0.059 infected bites per person and per night in October 2008 and October 2010 respectively. No transmission was observed between January and June 2009 (Figure 3).

**Table 1:** Main characteristics of *Anopheles *sampled during the longitudinal study

	Mosquitoes collected	Indoor/Total Endophagous rate%		Infected/total Infection rate%		Human blood meal/Total Anthropophagic rate%		L1014F kdr allele/total frequency%	
October 2008	784	132/331	39.88%	7/784	0.89%	164/181	90.61%	64/898	7.13%
January 2009	27	8/12	66.67%	0/27	0.00%	5/7	71.43%	0/38	0.00%
May 2009	161	30/40	75.00%	0/161	0.00%	31/43	72.09%	2/150	1.33%
October 2009	568	143/302	47.35%	6/568	1.06%	75/88	85.23%	77/608	12.66%
January 2010	73	14/26	53.85%	1/73	1.37%	16/21	76.19%	9/104	8.65%
		Chi2 = 21.5 p < .0001		Fisher exact p = 0.7		Fisher exact p < 0.01		Fisher exact p < 0.001	

*An. funestus*	21	1/1	100.00%	0/21	0.00%	5/10	50.00%	-	-
*An. arabiensis*	1055	139/313	44.41%	10/1055	0.95%	247/285	86.67%	103/1486	6.93%
*An. gambiae M*	30	4/6	67.67%	1/30	3.33%	9/12	75.00%	3/60	5.00%
*An. gambiae S*	128	15/41	36.59%	3/128	2.34%	30/32	93.75%	46/252	18.25%
*An. pharoensis*	278	141/251	56.18%	0/278	0.00%	0/1	0.00%	-	-
*An. wellcomei*	1	0/1	0.00%	0/1	0.00%	-	-	-	-
*An. ziemanni*	100	27/98	27.55%	0/100	0.00%	-	-	-	-
		Fisher exact p < 0.001		Fisher exact p = 0.09		Fisher exact p = 0.02		Chi2 = 36.6 p < 0.001	

Total	1613	327/711	45.99%	14/1613	0.87%	291/340	85.59%	152/1798	8.45%

Number, endophagous rate, infection rate, anthropophagic rate and L1014F kdr allele frequency according to season and species during the longitudinal study from October 2008 to January 2010. Global comparison by Pearson Chi^2 ^of Fisher exact test, p values are given.

Of the 711 specimens sampled by HLC, 327 (45.99%) were collected indoors and 384 (54.01%) outdoors. Significant variations in endophagous rate according to the season were observed (Chi^2 ^= 21.5, p < 0.001, Table 1) with lower values during rainy seasons. There was a significant difference in endophagous behaviour among the different species (Fisher exact p < 0.001, Table 1).

Of the 340 blood meal samples tested, 46 (13.53%) were from bovine or ovine origin (39 (84.78%) bovine, 7 (15.22%) ovine); 3 (0.88%) were mixed human-ovine or human-bovine and 291 (85.59%) were strictly of human origin. Significant variations in the anthropophilic rate were observed according to the season, with higher anthropophilic rates during the rainy season (Fisher exact p < 0.01, Table 1). Among species, there was a significant difference in anthropophagic rate (Fisher exact p = 0.002) with *An. arabiensis *and *An. gambiae *molecular form S being significantly more anthropophilic than *An. funestus *(Fisher exact p = 0.008 and 0.005 respectively).

Among *An. gambiae s.l*. specimens sampled, 899 were tested for the presence of kdr mutation. Homozygote L1014F kdr mutation genotype was identified in 15 specimens (1.7%), heterozygote L1014F kdr mutation in 122 (13.6%) and homozygote wild genotype in 762 (84.8%). L1014F kdr mutation allelic rate significantly changed according to the season (Fisher exact p < 0.001, Table 1) with higher values during the rainy seasons. L1014F kdr allelic frequency also varied according to the species (Pearson Chi2 = 36.6, p < 0.001) with the highest frequency observed in *An. gambiae *molecular form S.

## Discussion

The aim of this study was to identify and characterise potential malaria vector species in the middle Senegal River valley. A large variety of *Anopheles *was identified with seven sympatric species. While *An. gambiae *s.s. used to be the prevailing species in this region in the late 1990's [4], *An. arabiensis *was the most frequent species in this study and has been reported in all but one of the studied sites. It is known to be perfectly adapted to dry savannah areas where rainfall is low [17,18]. *An. gambiae *is known to be present and sympatric with *An. arabiensis *in relatively dry regions like the Niger River in Mali or Mauritania [19], especially in rice culture areas [20]. Both M and S molecular forms of *An. gambiae *s.s. were identified in this study. These two molecular forms are known to be excellent malaria vectors [21], although the S form seems to be more susceptible to *Plasmodium falciparum *than the M form [22]. The distribution of molecular forms M and S is influenced by environmental related factors and habitat characteristics. While *An. gambiae *M form needs permanent breeding sites provided by the presence of water such as in irrigation facilities or flooded areas [23,24], the S form is more strictly dependent on rainfall [25,26]. In this study, we observed the presence of both molecular forms S and M but only during rainy seasons. As previously reported in this region [5]*An. pharoensis *was identified in this study. *An. pharoensis *is known to be a potential malaria vector [6]. More surprising is the absence of *An. funestus *in most villages and its low density all the year long. Appearance and disappearance of *An. funestus *seems a complex phenomenon that is still unsolved even though rainfall changes seem to be the main explanation [8,27]. In other areas, *An. funestus *density has been shown to be subject to seasonal variations associated with rice growth periods [28].

During the cross-sectional study, *Anopheles *densities were extremely variable between villages, being relatively high in some of them. *Anopheles *density was not related to the proximity of the river but to other ecological conditions: it was higher in villages situated in flooded areas (Walo) than in villages with sandy ground (Dierry) and was correlated to the presence of ditch water for various activities, such as rice culture, gardening, brick manufacturing or animal watering. *Anopheles *density was therefore more related to local man-made conditions than to a large scale geographical context.

Blood meal identification is important in understanding vectorial capacity of malaria vectors and transmission dynamics [29]. *An. arabiensis *is known to have an opportunistic feeding behaviour [30]. Anthropophily, in areas where *An. arabiensis *predominates, may be high whether domestic animals are rare [31] or widely available [32]. *An. arabiensis *may also be highly zoophilic in other conditions [33,34]. In our study, *An. arabiensis *was highly anthropophilic, even though most of the householders kept cattle near their houses. Anthropophilic rates changed according to the season, with higher values in October, probably because most habitants of this region sleep outside during the hot season.

Although *Anopheles *density was high in some villages during the rainy season, CSP rate was low all the year round. Transmission only occurred during the rainy season and few months following. Similar infection rates have been reported in Dakar (Senegal) [35], Mauritania [36], Ethiopia [37] and Eritrea [38]. However, *An. arabiensis *sporozoite rates could be much higher in other villages or areas like Ndiop (Senegal) [39] or in Kenya [40]. Our data are in accordance with another study, indicating that ecological modifications of breeding sites influence mosquito density but not automatically malaria transmission [20]. As a result of the low transmission, malaria morbidity has been low in the Senegal River region in recent years (3 malaria attacks for 1000 inhabitants in 2009) [41].

The resistance of mosquitoes to DDT and pyrethroids, which have spread through sub-Saharan Africa during the last decades [42] has been recognized as being linked to the presence of knock-down resistance (kdr) mutation in *An. gambiae *s.l [10]. Although kdr mutation is only one among other mechanisms of resistance to insecticide, it is interesting to notice that it was present at low frequency in *An. arabiensis *and *An. gambiae *both molecular forms. Several studies have suggested that the use of agricultural pesticides favored the emergence and facilitated the spread of resistance within mosquito populations [43]. Although we did not specifically study this point, it is possible that changes in agricultural practice have contributed to the emergence of this resistance. A large scale implementation of insecticide treated nets (ITNs) is currently ongoing in Senegal [44]. The spread of resistance among mosquitoes could decrease the efficacy of this measure. On the other hand, the presence of ITNs can contribute to select resistant specimens as previously demonstrated in other areas [45].

## Conclusions

In conclusion, malaria transmission in the middle Senegal River basin is low, seasonal and maintained by *An. arabiensis*. Other vectors present in the area are *An. gambiae *M and S forms and *An. funestus*. The abundance of malaria vectors during the rainy season favors malaria epidemics. The situation calls for the improvement of vector control initiatives during the rainy season.

## Competing interests

The authors declare that they have no competing interests.

## Authors' contributions

JBS, FR, EH, LK and GR planned the study design. MON, SJB and LG performed field activity. MON and SS performed laboratory work. CM, MON analyzed the data, CM and CS drafted the manuscript. JFT and EH provided substantial improvement of the manuscript and CS and GR provided scientific supervision of the study. All authors approved the final version of the manuscript.
